# Surgical Improvement of Neuropathy-Induced Calf Muscle Hypertrophy and Creatine Kinase Elevation: A Case Report

**DOI:** 10.7759/cureus.66143

**Published:** 2024-08-04

**Authors:** Mamoru Fukuda, Yasufumi Ohtake, Yuma Hiratsuka, Tomoaki Ishizuka, Hirohiko Nakamura

**Affiliations:** 1 Neurological Surgery, Nakamura Memorial Hospital, Sapporo, JPN

**Keywords:** muscle pseudohypertrophy, calf muscle hypertrophy, nerve conduction studies, radiculopathy, peripheral neuropathy

## Abstract

Peripheral neuropathy and radiculopathy often result in skeletal muscle disorders, typically leading to muscle atrophy. Concurrent muscle hypertrophy or persistently elevated creatine kinase (CK) is rare. While muscle hypertrophy is commonly observed in myogenic diseases, such as muscular dystrophy, acromegaly, inflammatory myopathies, and hypothyroidism, reports of muscle hypertrophy caused by neuropathy are infrequent. We encountered a patient with persistently elevated CK levels and unilateral lower leg muscle hypertrophy associated with neuropathy. The patient had cauda equina syndrome symptoms and pain in the left lower leg. Lumbar spine magnetic resonance imaging (MRI) revealed central spinal stenosis, which was believed to be the cause of the symptoms. Lower-limb MRI revealed high signal intensity in the gastrocnemius muscle on fat-suppressed T2-weighted imaging. Surgical treatment improved the radiculopathy, hypertrophy, and pain in the left lower leg. During the one-year follow-up, improvement was confirmed with both MRI and nerve conduction studies. Calf muscle hypertrophy associated with neuropathy has been reported; however, no reports have demonstrated pre- and postoperative changes with MRI and nerve conduction studies. We report a patient with lower leg muscle hypertrophy and persistent CK elevation associated with neuropathy, along with a literature review.

## Introduction

Peripheral neuropathies and radiculopathies usually cause muscle atrophy. Muscle hypertrophy and persistently elevated creatine kinase (CK) are uncommon [1]. Muscle hypertrophy is frequently observed in myogenic disorders, such as muscular dystrophy, acromegaly, inflammatory myopathies, and hypothyroidism; however, reports of muscle hypertrophy due to neuropathy are limited. Surgery has been reported rarely in previous case reports, and many speculations remain unverified. There have been reports of calf muscle hypertrophy associated with neuropathy [1-4]. However, none have documented postoperative improvements in both CK levels and lower limb muscle hypertrophy. Additionally, no reports have described pre- and postoperative (longitudinal) changes in magnetic resonance imaging (MRI) and nerve conduction studies (NCS). We report a patient with lower leg muscle hypertrophy and persistent CK elevation associated with neuropathy. The patient underwent surgical treatment and showed improvement, strongly suggesting that the muscle hypertrophy resulted from the radiculopathy.

## Case presentation

A 66-year-old man began to experience intermittent claudication, numbness in the soles of both feet, and pain in his left lower leg approximately two years before the hospital visit, with the symptoms worsening progressively over time. One year earlier, he lost the ability to tiptoe on his left foot, resulting in difficulty walking. Six months earlier, he noticed an enlargement of his left lower leg. Elevated CK levels led to a suspicion of polymyositis, and he underwent further examination at the rheumatology department of another hospital. Although no elevations in thyroid hormones, liver enzymes, or autoantibodies were observed, he was referred to our neurology department on suspicion of myopathy.

**Figure 1:**
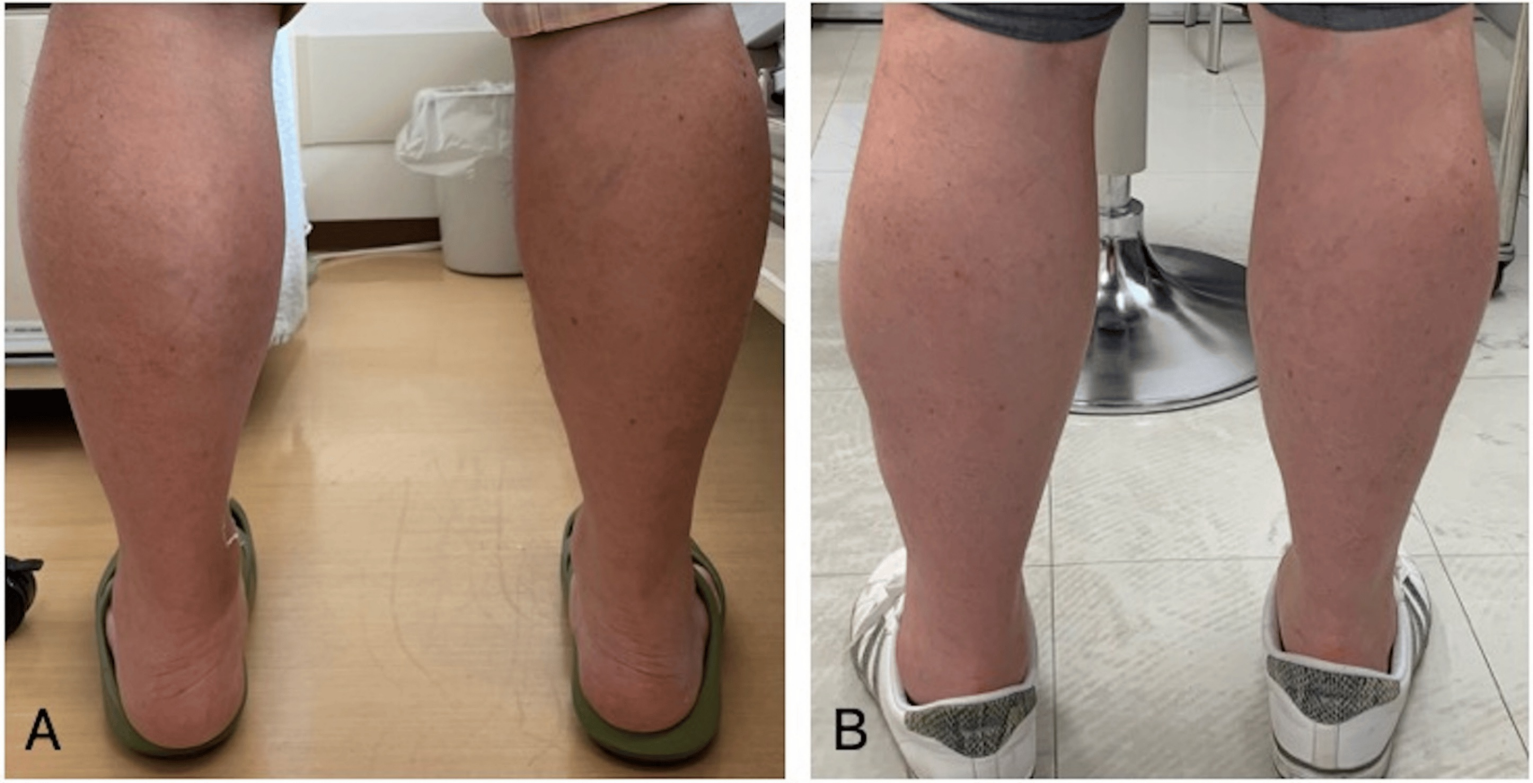
Bilateral changes in the calves. A: The measurements of the patient’s lower legs were 41.3 cm on the right and 43 cm on the left at the largest circumference, with visible swelling in the left lower leg and a noticeable difference between the left and right sides. B: At the one-year follow-up post-surgery, the measurements had improved to 40.0 cm on the right and 41.5 cm on the left.

On examination in our department, the patient’s muscle strength was decreased, with a left knee flexion manual muscle testing (MMT) grade 4, left ankle dorsiflexion MMT grade 3, and left ankle plantar flexion MMT grade 3. There was numbness from the dorsum of both lower legs to the soles of the feet. The patient required a cane for walking and exhibited intermittent claudication over approximately 200 m. Additionally, swelling was observed in the left lower leg, with a calf circumference of 41.3 cm on the right and 43 cm on the left, with a visible difference between the left and right side (Figure 1, Panel A).

**Figure 2:**
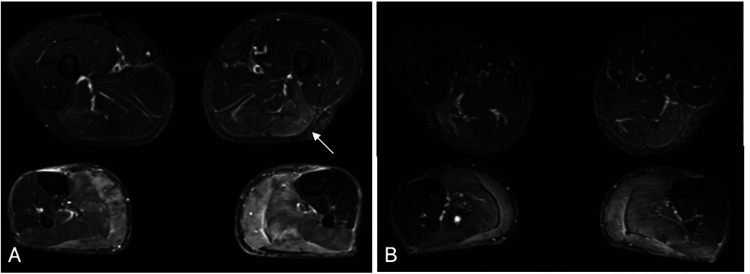
Pre- and postoperative changes in fat-suppressed T2-weighted MRI. A: Fat-suppressed T2-weighted image of the lower legs showing increased signal intensity primarily in the left biceps femoris and semitendinosus muscles (white arrow), with the left soleus muscle exhibiting a higher signal intensity compared with the right. B: One year postoperatively, the MRI showed that the increased signal intensity had improved in each area.

Fat-suppressed T2-weighted imaging of both lower legs revealed high signal intensity areas, predominantly on the left side in the biceps femoris and semitendinosus (Figure 2, Panel A, white arrow), bilateral gastrocnemius, and soleus muscles (Figure 2, Panel A).

**Figure 3:**
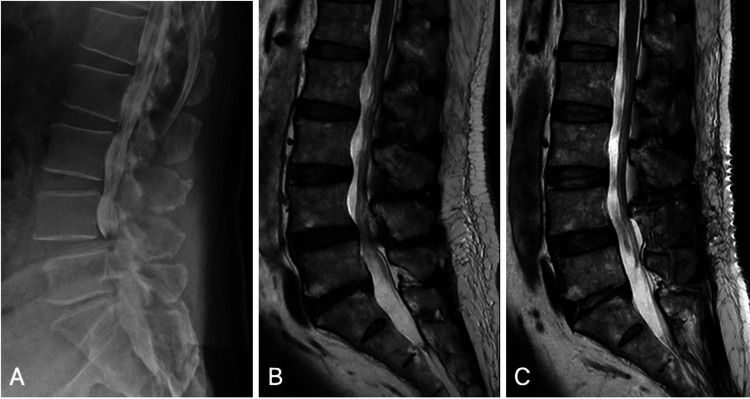
Preoperative myelography and changes in T2-weighted images before and after surgery. A: Myelography showing a total stop of the contrast agent at L4/5. B: MRI T2-weighted images showing central canal stenosis at the L4/5 level. C: Postoperative MRI showing resolution of the previous lumbar canal stenosis.

Lumbar spine X-rays showed no obvious instability; however, MRI revealed central canal stenosis at the L4/5 level (Figure 3, Panel A), and myelography showed a total stop of the contrast agent at the same level (Figure 3, Panel B). NCS revealed decreased compound muscle action potentials (CMAP) in the left tibial and right fibular nerves, with a particularly notable decreased CMAP in the left tibial nerve, at 3.45 mV. Electromyography (EMG) revealed a polyphasic pattern and high amplitude in the left gastrocnemius muscle.

MRI findings revealed areas of high signal intensity in the muscles innervated by the S1 nerve root. These observations were consistent with NCS and the myelography results, leading us to conclude a correlation between these findings. While the left calf presented with subjective symptoms and morphological changes, the findings in the right calf and left thigh were considered subclinical. Furthermore, no clear signs of myopathy were observed, and the changes were considered of neurogenic origin. Therefore, all findings were considered associated with lumbar canal stenosis.

Based on the physical examination and imaging findings, we concluded that the primary symptoms were due to central stenosis. There have been several reports of lower leg muscle hypertrophy due to S1 nerve root impingement [1-4]. In our patient, the EMG findings were consistent with a lesion of the left S1 nerve root. To definitively diagnose the condition, a biopsy of the gastrocnemius muscle was contemplated. However, as the predominant symptoms aligned with cauda equina syndrome, the patient declined to proceed with the biopsy. Laminectomy at L4/5 and hemilaminectomy at left L5/S1 were performed, as it was considered that these were the compressive lesions affecting the S1 nerve root. Both the intraoperative findings and postoperative MRI confirmed adequate decompression (Figure 3, Panel C). Sensory impairment in the left lower leg began to improve on the second postoperative day, and by the fourth postoperative day, the symptoms had completely resolved. By the seventh postoperative day, the patient was able to walk unaided, and he was discharged to his home on the 11th postoperative day.

The CK concentration was 609 U/L preoperatively and decreased to 233 U/L on the seventh postoperative day. MRI on the seventh postoperative day and one year later confirmed a gradual decrease in signal intensity in bilateral gastrocnemius and soleus muscles (Figure 2, Panel B). The circumference in the patient’s calves was 41.3 cm on the right and 43.0 cm on the left preoperatively and improved to 40.0 cm on the right and 41.5 cm on the left one year postoperatively (Figure 1, Panel B). Additionally, one year after surgery, NCS showed improvement in the CMAP in the right tibial nerve.

## Discussion

Neurogenic changes accompanying radiculopathy commonly result in muscle atrophy due to lower motor neuron damage [1]. Additionally, lower leg hypertrophy can occur in various pathological conditions, such as myositis, deep vein thrombosis, lymphedema, soft tissue tumors, and hypothyroidism [1]. Muscle hypertrophy in neuropathy is considered rare. This condition was first reported by Graves in 1848 as hypertrophy of the gastrocnemius muscle accompanying unilateral sciatic nerve pain [2]. In all reports of radiculopathies associated with muscle hypertrophy, S1 radiculopathy was observed in 86% of the patients, and increased serum CK was observed in 74% of the patients, indicating high frequencies [3]. However, there are also reports of hypertrophy of the tibialis anterior muscle due to L5 radiculopathy, and hypertrophy of the trapezius muscle on the same side occurred due to accessory nerve damage [4,5].

It is not easy to determine whether lower leg hypertrophy indicates muscle hypertrophy associated with radiculopathy. Signal changes in the lower leg muscles on MRI are nonspecific findings, and exclusion of other diseases is necessary. There have also been reports of lower leg muscle hypertrophy in peripheral nerve diseases, such as diabetic peripheral neuropathy and chronic inflammatory demyelinating polyneuropathy [6,7]. Lower leg hypertrophy may also be observed in conditions other than nerve diseases, and some reports consider this a pathological condition of focal myositis [8]. In such cases, the differential diagnoses comprise viral infections, such as influenza virus, coxsackieviruses, and cytomegalovirus; bacterial infections, such as Lyme disease and tuberculosis; fungal infections, such as candidiasis and aspergillosis; early symptoms of autoimmune diseases, such as Behcet’s disease, systemic lupus erythematosus, and Sjogren’s syndrome; and adverse effects of statin administration [9]. Generally, detailed neurological examination and evaluation of nerve compression findings on MRI and myelography are important in cases of leg hypertrophy with radiculopathy because of the presence of decreased muscle strength, sensory disturbances, and decreased tendon reflexes along the nerve root innervation area [10]. In the present case, high signal-intensity areas were observed in the left biceps femoris and semitendinosus muscles on T2-weighted imaging. Although the lesion was asymptomatic, signal changes on T2-weighted imaging were observed in both the right and left gastrocnemius muscles. As the nerves to the muscles in the thigh and lower leg that showed signal changes on MRI all originate from the S1 nerve root, S1 radiculopathy was considered a potential cause of the symptoms. Therefore, it was considered a priority to identify the compressive lesion affecting the S1 nerve root.

After an MRI examination, myelography was performed to confirm the central stenosis at L4/5, and a laminectomy at the same location was conducted. Additionally, to ensure decompression of the nerve root, a hemilaminectomy at L5/S1 was performed.

The mechanism by which nerve damage causes muscle hypertrophy is unclear. From previous reports [11,12], it can be inferred that there are two types of muscle hypertrophy. The first is true hypertrophy, where the muscle fibers themselves enlarge, and muscle biopsy shows a mixed pattern of enlarged muscle fibers with a large cross-sectional area and small atrophied muscle fibers [11]. The second type is pseudohypertrophy, where muscle fibers are replaced by other tissues, and muscle biopsy and MRI show muscle fibers replaced by adipose tissue and infiltration of inflammatory cells [7,13]. Two hypotheses for true hypertrophy and one hypothesis for pseudohypertrophy have been proposed. The first hypothesis for true hypertrophy is that this condition is induced by chronic muscle stimulation, based on EMG findings. Continuous stimulation by abnormal repetitive discharge in myokymia, seen as involuntary bundle muscle contractions, and myotonia, seen as muscle rigidity, promote true hypertrophy [10]. The second hypothesis is that, as a result of denervation and muscle atrophy in some muscles due to nerve root compression, the weight-bearing load is applied to some muscles that are reinnervated, resulting in true hypertrophy. Especially in the case of the muscles on the back of the lower leg, hypertrophy is likely because a strong load is continuously applied during daily activities, such as standing and walking [11]. The hypothesis for pseudohypertrophy is that the atrophied muscles are replaced by fat or that inflammatory cells infiltrate during the development of hypertrophy, causing inflammation and edema [13].

Persistently elevated CK may be associated with several mechanisms, such as fiber splitting, necrosis, and repeated regeneration during the development of muscle hypertrophy, as well as focal myositis induced by the infiltration of inflammatory cells, which elevates CK levels [3,12]. MRI signal changes in the lower leg muscles are believed to reflect inflammation and edematous changes occurring during hypertrophy [13]. However, these signal changes are observed not only in pseudohypertrophy but also in true hypertrophy. Therefore, the extent to which either true hypertrophy or pseudohypertrophy predominates varies by case. In the present case, muscle biopsy was not performed owing to the primary symptom of intermittent claudication. In such cases, it is possible to estimate the predominant cause using T1-weighted imaging and ultrasonography. Indeed, there have been reports of cases of fat degeneration confirmed by muscle biopsy, in which ultrasonography and T1-weighted imaging findings were consistent with fat degeneration of the lower leg muscles [1,6].

Regarding therapy for neurogenic symptoms accompanying lower leg muscle hypertrophy, conservative treatment and observation are often chosen if the symptoms are mild. Although there is no established treatment for moderate-to-severe cases, several approaches have been reported, including nerve root block [12], corticosteroid injection [14], and botulinum toxin injection [15]. Surgical interventions have been chosen in the presence of symptoms such as numbness or paralysis [16]. However, these cases are rare, and even when improvement in neurological symptoms is achieved, there may be no improvement in the hypertrophy of the gastrocnemius muscle [16]. Therefore, surgery is often performed to improve symptoms other than muscle hypertrophy. Notably, there are three reports of surgery for lumbar spinal stenosis induced by L5/S1 nerve root dysfunction with concurrent lower leg muscle hypertrophy, resulting in improvement of the muscle hypertrophy [6,17,18]. However, no reports have demonstrated postoperative improvement in both lower leg muscle hypertrophy and persistently elevated CK levels. In the present case, posterior decompression surgery resulted in a reduction in lower leg swelling, a decrease in CK levels, and a decrease in the signal intensity of the lower leg muscles on MRI. The improvement in CMAP during the postoperative NCS is considered to reflect the amelioration of lumbar spinal stenosis achieved through surgery. Reductions in signal intensity and CK levels were observed relatively early postoperatively and were considered to result from decreased inflammation and edema with rest. Therefore, it is believed that this case was mainly a case of pseudohypertrophy. Furthermore, as the nerve roots were decompressed with surgery, we hypothesized that the prevention of new denervation led to the cessation of muscle atrophy, necrosis, and fat degeneration, ultimately resulting in a reduction in the diameter of the lower leg muscles. Notably, regular MRI and NCS follow-ups were not performed in previously reported cases, whereas in the present case, improvement was confirmed in both examinations one year after treatment.

## Conclusions

Muscle hypertrophy and CK elevation due to neuropathy are rare, and many aspects of the underlying mechanism remain unclear. Muscle hypertrophy involves two mechanisms: true hypertrophy, which is the enlargement of muscle fibers, and pseudohypertrophy, which is the replacement of muscle fibers by other tissues, each with different hypotheses regarding the pathogenesis. Persistently elevated CK levels are considered linked to mechanisms such as fiber splitting, necrosis, and repeated regeneration during muscle hypertrophy, as well as focal myositis induced by inflammatory cell infiltration. In this report, posterior decompression surgery led to improvement of calf muscle hypertrophy and elevated CK levels, with postoperative MRI and NCS findings confirming the improvement one year after surgery.
